# Exploring the role of clan culture in promoting nurses’ green behaviors: paternalistic leadership as a mediator and workplace loneliness as a moderator

**DOI:** 10.1186/s12912-025-03027-7

**Published:** 2025-04-17

**Authors:** Hanan Meslhy Mohamed, Manal Saleh Moustafa Saleh, Nora Mahdy Attia, Manal Mohammed Ahmed Abdelaziz, Azza Abdeldayem Ata

**Affiliations:** 1 Nursing Administration, Faculty of Nursing, Zagazig University, Zagazig, Egypt; 2 Department of Nursing Sciences, College of Applied Medical Science, Shaqra University, Shaqra, Saudi Arabia; 3 Nursing Department, North Private College of Nursing, Arar, Saudi Arabia; 4 Nursing Administration, Faculty of Nursing, Tanta University, Tanta, Egypt

**Keywords:** Clan culture, Green behavior, Paternalistic leadership, And workplace loneliness

## Abstract

**Background:**

Clan culture is characterized by a family-like environment that emphasizes collaboration, support, and a sense of belonging among employees. Paternalistic leadership combines strong authority with benevolence, where leaders act as parental figures, guiding and caring for their subordinates. In organizations with a clan culture, paternalistic leadership can thrive, as both prioritize close-knit relationships and employee well-being. This alignment can foster a supportive work environment, enhancing job satisfaction and reducing feelings of isolation among employees.

**Aim:**

This study seeks to investigate the impact of clan culture on nurses’ green behavior, with a specific emphasis on how paternalistic leadership operates as a mediator and workplace loneliness as a moderator.

**Subject and methods:**

780 nurses from the Zagazig University Hospitals in Zagazig City, Egypt, were chosen at a systematic random sampling method. This study used a descriptive correlational design. Four instruments were employed to collect the data: The paternalistic leadership scale, the employee green behaviors descriptive norms scale, the clan culture scale, and the revised UCLA loneliness scale.

**Results:**

Findings revealed that clan culture has a significant negative direct effect on green behaviors; nevertheless, it positively influences paternalistic leadership. Paternalistic leadership positively affects green behaviors. Paternalistic leadership partially mediates the relationship between clan culture and green behaviors. Workplace loneliness moderates the relationship between clan culture and green behaviors.

**Conclusion:**

This study underscores the importance of leadership in translating organizational culture into sustainable practices. While clan culture, on its own, may deprioritize green behaviors, paternalistic leadership serves as a critical mediator, enabling organizations to align relational harmony with environmental sustainability. By fostering supportive leadership and integrating sustainability into cultural values, organizations can address both relational and ecological goals.

**Implications for nursing management and leadership:**

To address these findings, organizations should prioritize enhancing clan culture while avoiding its potential drawbacks, promote benevolent leadership while reducing authoritarian tendencies, and implement targeted strategies to improve green behavior and mitigate workplace loneliness. Interventions could include leadership training, employee support programs, and environmental awareness campaigns. Also, the findings highlight the need for a holistic approach to organizational development. Strategies to enhance clan culture and paternalistic leadership should incorporate sustainability and inclusivity as core values. Specific interventions include leadership training programs, sustainability workshops, and initiatives to foster social connections among nurses.

**Clinical trial number:**

Not applicable.

## Introduction

Most of the work in the healthcare industry is still done by nurses, despite the advancements in technology. Because of the high degree of expertise and the intense nature of human labour, human resources are crucial in this industry. For health workers to operate successfully and efficiently, job satisfaction and motivation must be guaranteed. Employees may, however, occasionally display unfavourable attitudes toward their jobs that affect their motivation and job satisfaction. In health organizations, such actions may result in unintended negative outcomes or a decline in service quality. Conversely, the strong sense of trust inherent in clan culture allows teams to function cohesively, improving decision-making and patient safety [1].

Clan culture refers to an organizational culture characterized by a family-like, collaborative environment that emphasizes teamwork, shared values, and interpersonal relationships among nursing staff [2]. This type of culture enhances job satisfaction and emotional well-being, addressing challenges like burnout and turnover that are prevalent in nursing. By promoting a sense of belonging and community, it reduces feelings of isolation and builds resilience among healthcare professionals. Nurses in a clan-oriented workplace are more likely to be engaged, motivated, and committed to organizational goals, including adopting innovative practices and sustainability initiatives [3].

Clan culture promotes collaboration, support, and shared goals, creating an environment where sustainability efforts can thrive. When combined with green behavior, it can drive meaningful, eco-friendly improvements in healthcare practices [4]. Green behavior encompasses sustainable practices that nurses adopt to reduce healthcare’s environmental impact. This includes minimizing waste, conserving energy, using resources efficiently, and promoting eco-friendly initiatives. Such actions support the broader goals of sustainable healthcare and environmental stewardship [3].

Leadership significantly influences employees’ green behavior, with different leadership styles shaping pro-environmental actions in organizations. Managers play a key role in preventing undesirable behaviors. In paternalistic leadership, leaders act as parental figures, providing guidance and support while fostering a sense of responsibility among subordinates [5].

Paternalistic leadership is a leadership style that combines authority with a paternal-like concern for employees. Leaders in this framework take on a role similar to that of a parent, prioritizing the well-being of their team members while maintaining control over decision-making [6]. Paternalistic leaders often exhibit care and concern for their employees, which aligns well with the nurturing environment of clan culture. This creates a workplace where employees feel valued and supported, leading to higher job satisfaction and loyalty. While paternalistic leadership involves an authoritative figure, the benevolent aspect fosters a familial atmosphere typical of clan culture. Leaders guide their teams while also providing emotional and professional support [7].

Paternalistic leadership combines strong authority, supportive guidance, and ethical conduct. It consists of three key dimensions: Authoritative, where leaders exercise firm control and expect compliance; benevolent, where leaders care for employees’ well-being in a nurturing manner; and moral, where leaders uphold ethical standards and serve as role models for integrity within the organization [5].

Clan culture can play a significant role in addressing work loneliness, which is a common issue faced by many healthcare professionals. Workplace loneliness refers to the subjective experience of feeling emotionally and socially disconnected from colleagues and the work environment. This sense of isolation can lead to decreased job satisfaction, increased stress, and diminished overall well-being among nurses. Workplace loneliness has been associated with factors such as limited social interaction with leaders, reduced trust in leadership, and a lack of meaningful work. Addressing workplace loneliness in nursing is crucial for maintaining a healthy work environment and ensuring high-quality patient care. Strategies to mitigate this issue include fostering open communication, building supportive relationships among staff, and creating opportunities for social interaction and professional development [4]. While direct research linking clan culture to green behavior is limited, the collaborative and supportive nature of clan cultures can encourage collective efforts toward sustainability. The emphasis on shared values and community within a clan culture may promote organizational initiatives that support green behaviors among employees [8].

This study seeks to investigate the impact of clan culture on nurses’ green behavior, with a specific emphasis on how paternalistic leadership operates as a mediator and workplace loneliness as a moderator.

### Literature review and hypothesis development

Clan culture is one of the four organizational culture types in the competing values framework. Clan culture fosters employee engagement and commitment by emphasizing trust, open communication, and shared goals. Employees feel valued and empowered, leading to greater motivation to align with the organization’s values, including sustainability initiatives and green behavior [9]. In addition, Social Exchange Theory: Paternalistic leadership relies heavily on social exchange, when leaders who demonstrate genuine concern for employees’ well-being can foster a sense of loyalty and responsibility. This relationship encourages employees to engage in green behaviors to support the organization’s sustainability goals [10].

Paternalistic leadership, characterized by authority and benevolence, can significantly influence employee green behavior (EGB). *Benevolent leadership*: fosters a supportive environment, enhancing employees’ psychological ownership, a state where employees feel like they own the organization. This sense of ownership motivates employees to engage in behaviors that benefit the organization, including green initiatives. *Authoritarian leadership*: may negatively impact employees’ organization-based self-esteem (OBSE), the degree to which employees believe they are valued members of the organization. Lower OBSE can reduce motivation to engage in discretionary behaviors like EGB [11].

Paternalistic leaders, who emphasize ethical behaviour and long-term responsibility, are likely to promote green behaviour by framing environmental sustainability as part of their moral duty to future generations. In such an environment, employees are motivated not only by internal values but also by loyalty to the leader and the organization. Leaders in paternalistic cultures are often positioned as role models who can inspire employees to adopt green practices and integrate sustainability into daily work life, with an emphasis on the well-being of society and the environment [12].

Loneliness refers to the subjective feeling of being disconnected or isolated from others, despite the presence of people around. It is a psychological state often linked to social relationships, where individuals feel that their social needs are unmet [13]. In the workplace, loneliness can be particularly problematic, leading to decreased job satisfaction, lower motivation, and poor mental health. Self-determination theory suggests that social environments that foster relatedness—the need to feel connected to others—can mitigate loneliness and promote motivation. The theory emphasizes the importance of connecting others as a basic psychological need so employees who feel connected to their peers and leadership are more likely to adopt sustainable practices because they feel part of a collective effort to improve the environment [12].

Clan culture is characterized by a focus on collaboration, teamwork, and values such as care, trust, and shared responsibility. In the context of healthcare, this culture can create an environment where sustainability is not just an individual effort but part of the collective ethos of the organization. Nurses operating within a clan culture may feel more empowered and supported to engage in green behaviors because of the collaborative and supportive nature of their workplace. This can include promoting environmentally friendly practices among colleagues and patients. As well, Nurses in organizations with a strong clan culture are more likely to see sustainable practices as an ethical duty aligned with their professional values [14].

The integration of clan culture and paternalistic leadership in nursing creates a supportive and collaborative environment that benefits both nurses and patients. By fostering strong relationships and prioritizing employee well-being, nursing leaders can enhance job satisfaction, reduce turnover, and ultimately improve patient care outcomes. Understanding these dynamics is crucial for advancing nursing practice in today’s healthcare settings. A strong clan culture supported by paternalistic leadership helps build resilience among nursing teams, allowing them to cope better with the demands of the job. Also, when nurses feel cared for and valued within a clan culture, they are less likely to leave the organization, which is vital in addressing nursing shortages [15]. The nurturing environment of clan culture, coupled with the support of paternalistic leaders, can lead to higher job satisfaction among nurses [5]. Therefore, the study proposed the following hypotheses:

#### H1

***Clan culture influences nurses’ green behavior***

#### H2

***Clan culture influences paternalistic leadership***

A paternalistic approach fosters strong relationships among team members, creating a sense of community. This cohesion can enhance collective efforts toward green behaviour, as nurses’ work together on sustainability initiatives [16]. Leaders who demonstrate commitment to environmental sustainability can inspire their teams. By modelling green behaviour, paternalistic leaders can influence nurses to adopt similar practices in their work. By fostering an environment of trust and empowerment, nurse leaders can not only promote environmental sustainability but also enhance team morale and job satisfaction. Understanding this dynamic is crucial for advancing both nursing practice and environmental responsibility in healthcare [17]. As a result, this study suggested the hypothesis that follows:

#### H3

***Paternalistic leadership influences nurses’ green behavior***

Paternalistic leadership, often characterized by leaders who take a fatherly or protective approach to managing employees (offering guidance, care, and support), could influence how employees perceive and enact behaviors such as environmental sustainability, especially within the context of a clan culture that values teamwork, collaboration, and shared responsibility. Paternalistic leadership might act as a mediator between clan culture and nurses’ green behavior by: Encouraging and reinforcing the importance of green behavior through direct guidance and mentorship, providing emotional and moral support to nurses to reduce any resistance or obstacles to adopting sustainable practices, and aligning nurses’ values with organizational goals related to sustainability [18].

Paternalistic leadership mediates the relationship between clan culture and employee green behavior by reinforcing shared values, enhancing psychological ownership, influencing organization-based self-esteem, and promoting a supportive organizational climate. These mechanisms collectively foster an environment where employees are motivated to engage in behaviors that support environmental sustainability [6]. Consequently, the study formulated the following hypothesis:

#### H4

***Paternalistic leadership mediates the relationship between the clan culture and nurses’ green behavior***

Work loneliness can reduce employees’ sense of connection and commitment to organizational values and can hinder nurses’ willingness to engage in green behaviours, which may undermine the positive effects of clan culture on green behaviours [19]. In healthcare settings, where clan culture is intended to foster collaboration, trust, and shared responsibility, work loneliness may moderate the extent to which nurses engage in environmentally sustainable practices. Loneliness at work weakens the effect of a supportive and collaborative organizational culture, making it more difficult for nurses to feel motivated to engage in green behaviours. However, low loneliness can enhance the positive effects of clan culture, leading to stronger pro-environmental engagement [5]. As a result, the research proposed the following hypothesis:

#### H5

***Work loneliness moderates the relationship between the clan culture and nurses’ green behaviors***

Recent literature underscores a significant gap in research exploring the integration of clan culture, paternalistic leadership, workplace loneliness, and employee green behavior. While individual studies have examined these constructions separately or in limited combinations, comprehensive analyses encompassing all four variables remain scarce. There is a notable gap in research specifically examining the integration of clan culture, a family-like, collaborative organizational environment, and green behavior. For example, A 2024 study investigated the role of green organizational culture (GOC) in promoting employee green behavior (EGB), highlighting the mediating effects of perceived organizational support and green organizational identity. The findings suggest that while GOC can indirectly foster EGB, the direct influence of specific cultural types, such as clan culture, on green behavior remains underexplored [20].

Furthermore, A 2024 analysis of employee green behavior research trends emphasized that while individual characteristics and organizational green culture significantly influence green behavior, there is a lack of discussion on how specific cultural frameworks, such as clan culture, affect sustainable development within organizations [1]. Moreover, several studies have investigated the impact of paternalistic leadership on employee green behavior (EGB). For instance, a study explored how benevolent and authoritarian dimensions of paternalistic leadership affect EGB, identifying psychological ownership as a mediating factor. The findings suggest that while paternalistic leadership influences EGB, the specific integration with clan culture remains underexplored [11].

**Fig. 1:**
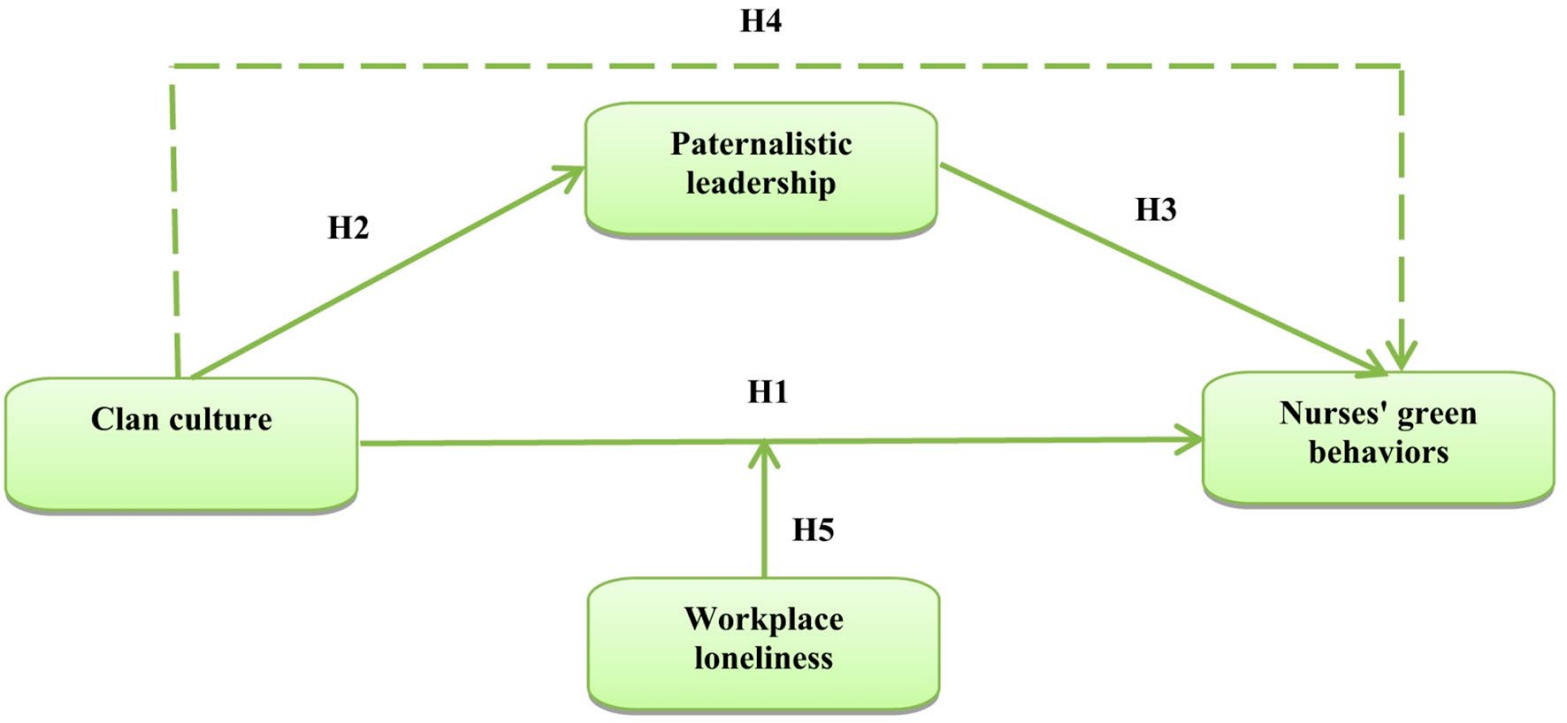
The conceptual model of the study

Furthermore, although previous research emphasizes the significance of clan culture and paternalistic leadership in enhancing nurses’ sustainable and green behaviours, insufficient empirical research has been done to examine the mediating role of paternalistic leadership and the moderating role of work loneliness in the relationship between clan culture and nurses’ green behaviour. Addressing this gap could provide actionable insights to improve sustainability efforts and emotional well-being in healthcare settings. Thus, this study aimed to fill this gap by investigating the interrelationships among clan culture, green behavior, paternalistic leadership, and workplace loneliness among nurses, with a specific emphasis on how paternalistic leadership operates as a mediator and workplace loneliness as a moderator (Fig 1).

### The study objective

This study seeks to investigate the impact of clan culture on nurses’ green behavior, with a specific emphasis on how paternalistic leadership operates as a mediator and workplace loneliness as a moderator.

## Methodology

### Study design

The purpose of this study was achieved using a descriptive correlational research design. According to [21] correlational design was employed to characterize variables and investigate correlations between them.

### Study setting

The study was conducted at Zagazig University Hospitals (academic hospitals), Al Sharqia, Egypt, which include two sectors involving nine teaching hospitals. The total bed capacity of the hospitals is 2043 beds.

### Participants and sample size

This study adopted a proportionate stratified random sampling procedure which according to [22] is appropriate for a heterogeneous population. Stratified random sampling involves dividing the entire heterogeneous population into homogeneous subgroups called strata; and from each stratum, the units are randomly sampled to represent the whole population. This study therefore proportionately determined the number of nurses in each stratum to be considered. A proportionate stratified random sample was determined using an open-source sample size calculator that is available at https://www.calculator.net/sample-size-calculator. to establish the required sample size by computing the overall size of the population of the prior all hospitals (Zagazig University Hospitals). The required sample size was determined at confidence level = 95%, margin of errors = 5.0%, population proportion = 50%, and total population size = 2770 nurses. After accounting for a 15% dropout rate, the required sample size was 780 staff nurses who were licensed, on duty during the study period, and had at least one year of experience at their present hospital to assure familiarity with workplace dynamics.

Once the researchers collected the needed number of nurses, at the first stage they divided the heterogeneous population of Zagazig University Hospitals into nine homogeneous strata. These strata include New Surgical Hospital, Internal Medicine Hospital, Emergency Hospital, Gynecology and Obstetrics Hospital, Out-Patient Hospital, Cardiac and Chest Hospital, El-Salam Hospital, Pediatric Hospital, and El-Sadat Hospital. At the second stage, in each stratum a sample of each hospital to be considered in the study was determined according to the following formula: Number of nurses in each hospital X the required sample size / Total number of nurses in all hospitals. Finally, the number of respondents from each sampled hospital was calculated and randomly selected. This sampling procedure ensured better coverage of the target population because each stratum was properly represented within the sample [23] (Table 1).

**Table 1:** Required sample size of nurses from each hospital

Hospital name	Total number of nurses	Required sample size
1. New–Surgical Hospital	551	156
2. Internal Medicine Hospital	523	147
3. Emergency Hospital	345	97
4. Gynecology and Obstetrics Hospital	202	57
5. Out-Patient Hospital	189	53
6. Cardiac and Chest Hospital	357	100
7. El-Salam Hospital	295	83
8. Pediatric Hospital	262	74
9. El-Sadat Hospital	46	13

### Instruments used in the study

The data for this study were obtained using four standardized scales.

#### Clan culture scale

It is based on the Organizational Culture Measurement Scale of [24] The scale included six statements to measure nurses’ perception regarding clan culture. The elements of the measure were rated using a five-point Likert scale ranging from 1 (strongly disagree) to 5 (strongly agree). The internal consistency reliability of the scale was assessed using Cronbach’s alpha coefficient, which was found to be 0.78.

#### Employee green behavior descriptive norms scale

It was developed by [25] to assess the level to which staff nurses experience descriptive norms regarding green behaviour. It included 40 items divided into five categories: Working sustainability (9 items), avoiding harm (7 items), conserving (10 items), influencing others (6 items) and taking initiative (8 items). Every item on the scale was rated using a five-point Likert scale ranging from 1 = strongly disagree to 5 = strongly agree. Cronbach’s alpha was as follows: Working sustainably (α = 0.87), avoiding harm (α = 0.73), conserving (α = 0.77), influencing others (α = 0.74), and taking initiative (α = 0.69). The scale’s internal consistency reliability for the total scale was 073.

#### Paternalistic leadership scale

It was developed by [26] to assess the perceptions of the participants about paternalistic leadership. It contains 26 items subdivided into three dimensions, namely: Benevolent leadership (11 items), moral leadership (6 items), and authoritarian leadership (9 items). Every item on the scale was rated using a five-point Likert scale that went from 1 = strongly disagree to 5 = strongly agree. Cronbach’s Alpha coefficients based on the reliability findings for the three dimensions were 0.84 for the benevolent leadership factor, 0.95 for the moral leadership factor, and 0.87 for the authoritarian leadership factor. The internal consistency reliability for the total scale was 0.89.

#### Revised UCLA loneliness scale

This scale was developed by [27] to assess the level of workplace loneliness among individuals. It consists of 20 statements, ten items of which were positive worded and others were negative worded which reflected how lonely individuals described their experience. Responses to the scale were measured on a four-point Likert scale ranging from 1 (never) to 4 (always). The internal consistency and reliability of the scale were assessed using Cronbach’s alpha coefficient, which was found to be 0.94.

#### The demographic information of study participants

The demographic information of the participants was collected, including gender, age, education, department, years of experience, and marital status.

### Validity of the tools

To guarantee that the participating nurses, whose native language is Arabic, comprehended the study measures, all items were translated into Arabic. A panel of five university professors proficient in both Arabic and English reviewed the translation for accuracy. Additionally, another panel of five bilingual university professors conducted a back-translation from Arabic to English to ensure consistency. To evaluate content and face validity, seven nursing professors specializing in nursing administration carefully examined the measures. The panelists affirmed that the measures were suitable for this study, and no modifications were necessary based on their assessments. The factor loadings were deemed satisfactory, with values exceeding 0.70, confirming the validity of the questionnaire’s dimensional structure.

### Pilot study

A pilot study was conducted to evaluate the questionnaires’ comprehensibility and the amount of time needed to complete them. To determine the required sample size, a preliminary mean of the outcome variables was also computed. The final study sample did not contain the 78 nurses (10% of the sample size) who were chosen for the pilot study from participating hospitals. The pilot nurses attested to the questionnaire’s clarity and understandability, and it took from 20 to 45 min to complete.

### Data collection

Data for this study was collected through self-reported assessments from staff nurses between the end of June to the end of August 2024. Before data collection, all necessary ethical approvals were obtained. During an initial meeting, each unit’s head nurse was informed about the study’s objectives and asked to support the data collection process. Nurses who met the inclusion criteria were provided with sealed envelopes containing the survey questionnaires to ensure perceived anonymity and reduce response bias. These envelopes were distributed at their respective workstations during shifts. A cover letter accompanying the questionnaire explained the purpose of the study, assured participants that their responses would be anonymous and voluntary, and provided instructions to reflect on their experiences with a specific nurse manager. Participants were asked to return the completed questionnaires in sealed envelopes to a designated drop box located in a common area of each unit by a specified deadline, ensuring confidentiality and minimizing any pressure to respond. The voluntary completion and submission of the surveys indicated an agreement to participate. This approach aimed at minimizing direct interaction between the research team and participants, fostering a private and neutral environment that encouraged honest responses.

### Ethical considerations and consent to contribute

The Ethics Committee of the Faculty of Nursing, Zagazig University, granted ethical approval for the study (reference number: ZU.Nur REC#: 263). All relevant information about the study was provided in the first section of the consent form. The questionnaire included a statement outlining the aim and nature of the study. Participants were asked to indicate their agreement to give informed consent before starting the survey. They were assured of the privacy and confidentiality of their responses, the voluntary nature of their participation, and that their involvement or absence would not affect their grades or result in any negative consequences. This ensured that participants could freely decide to participate without concerns of work repercussions. Participants provided informed consent by the criteria outlined in the Helsinki Declaration and were informed that they had the right to withdraw from the study at any time. This right to withdraw was communicated both verbally and in writing at the beginning of the study. The authors confirm that all methods were performed according to the relevant guidelines and regulations.

### Statistical design

For data analysis, IBM SPSS 25 and AMOS 26 were utilized. Descriptive statistics were used to present the study variables and the nursing characteristics. The independent t-test and analysis of variance (ANOVA) were used to evaluate differences in clan culture, green behaviors, paternalistic leadership, and workplace loneliness regarding the characteristics of the study sample. The bivariate correlations between the research variables were investigated using Pearson’s correlation. A structural equation model (SEM) was employed to explore the direct and indirect effects within the proposed conceptual framework. Specifically, the mediation effect of paternalistic leadership on the relationship between clan culture and green behaviors was tested. The statistical significance of the indirect effect was tested using the bootstrapping method in AMOS to assess the significance of the mediation. To ensure the robustness of the structural equation model (SEM), we indeed assessed model fit using several standard indices, including the Comparative Fit Index (CFI), Tucker-Lewis Index (TLI), Root Mean Square Error of Approximation (RMSEA), and Standardized Root Mean Square Residual (SRMR). Our model yielded a CFI of 0.94, a TLI of 0.93, an RMSEA of 0.047, and an SRMR of 0.06, which align with the commonly accepted thresholds for a good model fit. To ensure the reliability and validity of the study instruments, Cronbach’s alpha was calculated to validate the measurement model and confirm the distinctiveness of the study constructs. P values with two tails < 0.05 indicated statistical significance and p-value with two tails < 0.01 indicated high statistical significance.

## Result

Table 2 presents the relationship between demographic characteristics and different study variables. The majority of contributors (57.8%) were aged 35 years or younger, with most being female (50.8%) and single (58.7%). Additionally, 55.9% had 10–20 years of experience, and 57.8% held a nursing institute qualification. Moreover, 81.4% were employed in medical-surgical departments. Gender and marital status exhibited significant differences, with male and married nurses demonstrating higher perceptions of clan culture compared to their female and single counterparts (*p* < 0.01). Additionally, educational background and department impacted clan culture, as nurses with a Bachelor of Nursing and those working in medical-surgical departments scored the highest (*p* < 0.01).

Moreover, nurses’ perceptions of paternalistic leadership varied significantly across all demographic characteristics, except for their department (*p* < 0.01). Similarly, nurses’ green behavior showed statistically significant differences based on their demographic characteristics (*p* < 0.01). This suggests that individual traits may play a role in promoting environmentally friendly practices in the workplace. Although loneliness showed only slight variations across groups, the most notable differences were found in gender (*p* < 0.01) and marital status (*p* < 0.05); with female and married nurses reporting higher levels of loneliness.

Table 3 investigates the correlation coefficients between the studied variables. As observed from the table, there was a significant and positive correlation between clan culture and paternalistic leadership (*r* = 0.399, *p* < 0.01). Conversely, clan culture correlates negatively with green behavior (*r* = − 0.378, *p* < 0.01), and workplace loneliness (*r* = − 0.267, *p* < 0.01). These results highlight that clan culture fosters leadership styles emphasizing interpersonal care (paternalistic leadership) and reduces workplace loneliness through its supportive and relational focus. However, it may also unintentionally deprioritize green behavior, reflecting a potential trade-off between relational harmony and broader organizational goals like environmental sustainability.

**Table 2:** Nurses’ demographic features and differences in the research variables (*n* = *780)*

Characteristic	Category	No. (%)	Clan culture		Paternalistic leadership		Green behavior		Loneliness	
			M (SD)	t/F (p)	M (SD)	t/F (p)	M (SD)	t/F (p)	M (SD)	t/F (p)
Age (years)^a^	≤ 35	451 (57.8)	25.53 (3.45)	*t* =- 1.851 (0.06)	87.51 (5.92)	*t* = 2.374 (0.01) **	134.21 (8.43)	*t* = − 2.792 (0.005) **	60.96 (5.97)	*t* = − 0.698 (0.48)
	> 35	329 (42.2)	25.09 (3.24)		86.58 (4.99)		136.04 (9.42)		61.24 (5.27)	
Gender ^a^	Male	384 (49.2)	25.86 (2.65)	*t* = 4.259 (0.000) **	87.65 (5.14)	*t* = 2.617 (0.009) **	132.22 (7.08)	*t* = − 8.986 (0.000) **	60.31 (5.62)	*t* = − 3.743 (0.000) **
	Female	396 (50.8)	24.85 (3.88)		86.61 (5.92)		137.77 (9.65)		61.82 (5.65)	
Marital status ^a^	Single	458 (58.7)	24.39 (3.49)	*t* = − 10.45 (0.000) **	86.31 (4.16)	*t* = − 4.562 (0.000) **	135.92 (9.37)	*t* = 3.623 (0.000) **	60.70 (6.17)	*t* = − 2.329 (0.02) *
	Married	322 (41.3)	26.71 (2.66)		88.28 (6.95)		133.65 (8.03)		61.62 (4.86)	
Experience (years) ^b^	< 10 years	156 (20.0)	25.39 (0.68)	*F =* 2.431 (0.08)	85.98 (6.26)	*F =* 15.60 (0.000) **	131.58 (9.47)	*F =* 16.45 (0.000) **	61.13 (6.92)	*F =* 0.169 (0.84)
	10–20 years	436 (55.9)	25.14 (0.97)		88.09 (5.44)		136.25 (7.91)		61.15 (5.57)	
	> 20 years	188 (24.1)	25.78 (2.82)		85.82 (4.77)		134.87 (9.86		60.87 (4.77)	
Education ^b^	Nursing diploma	80 (10.3)	25.60 (3.55)	*F =* 25.29 (0.000) **	86.50 (7.18)	*F =* 20.85 (0.000) **	131.80 (9.18)	*F =* 5.88 (0.003) **	62.30 (4.46)	*F =* 2.74 (0.06)
	Nursing institute	451 (57.8)	24.67 (3.74)		86.22 (5.54)		135.22 (9.19)		60.75 (6.23)	
	Bachelor of nursing	249 (31.9)	26.49 (2.01)		88.95 (4.51)		135.56 (8.09)		61.27 (4.89)	
Department ^a^	Medical and surgical	635 (81.4)	25.69 (3.14)	*t* = 5.413 (0.000) **	87.29 (5.56)	*t* = 1.766 (0.07)	134.54 (8.94)	*t* = − 2.967 (0.003) **	60.89 (5.67)	*t* = − 1.879 (0.06)
	Critical care units	145 (18.6)	23.82 (3.88)		86.39 (5.56)		136.89 (8.50)		61.88 (5.67)	

M = Mean. SD = Standard deviation. *(a)* Analyzed by independent t-test. (b) Analyzed by one way ANOVA * p is significant ˂ 0.05 level (2-tailed). ** p is significant ˂ 0.01 level (2-tailed)

Similarly, paternalistic leadership demonstrated a significant positive association with nurses’ green behavior (*r* = 0.386, *p* < 0.01) and a negative significant association with loneliness (*r* = − 0.394, *p* < 0.01). These results highlight the dual benefits of paternalistic leadership. It not only encourages nurses to engage in green, sustainable practices by fostering a sense of responsibility and ethical awareness but also addresses psychosocial challenges like loneliness by creating a supportive and inclusive work environment.

Moreover, nurses’ green behavior showed a negative correlation with workplace loneliness (*r* = − 0.185, *p* < 0.01). This result indicates that nurses who experience lower levels of workplace loneliness are more likely to engage in green behavior, which includes environmentally sustainable practices. In contrast, those who feel lonelier in the workplace may be less motivated or less inclined to participate in such behaviors.

**Fig. 2:**
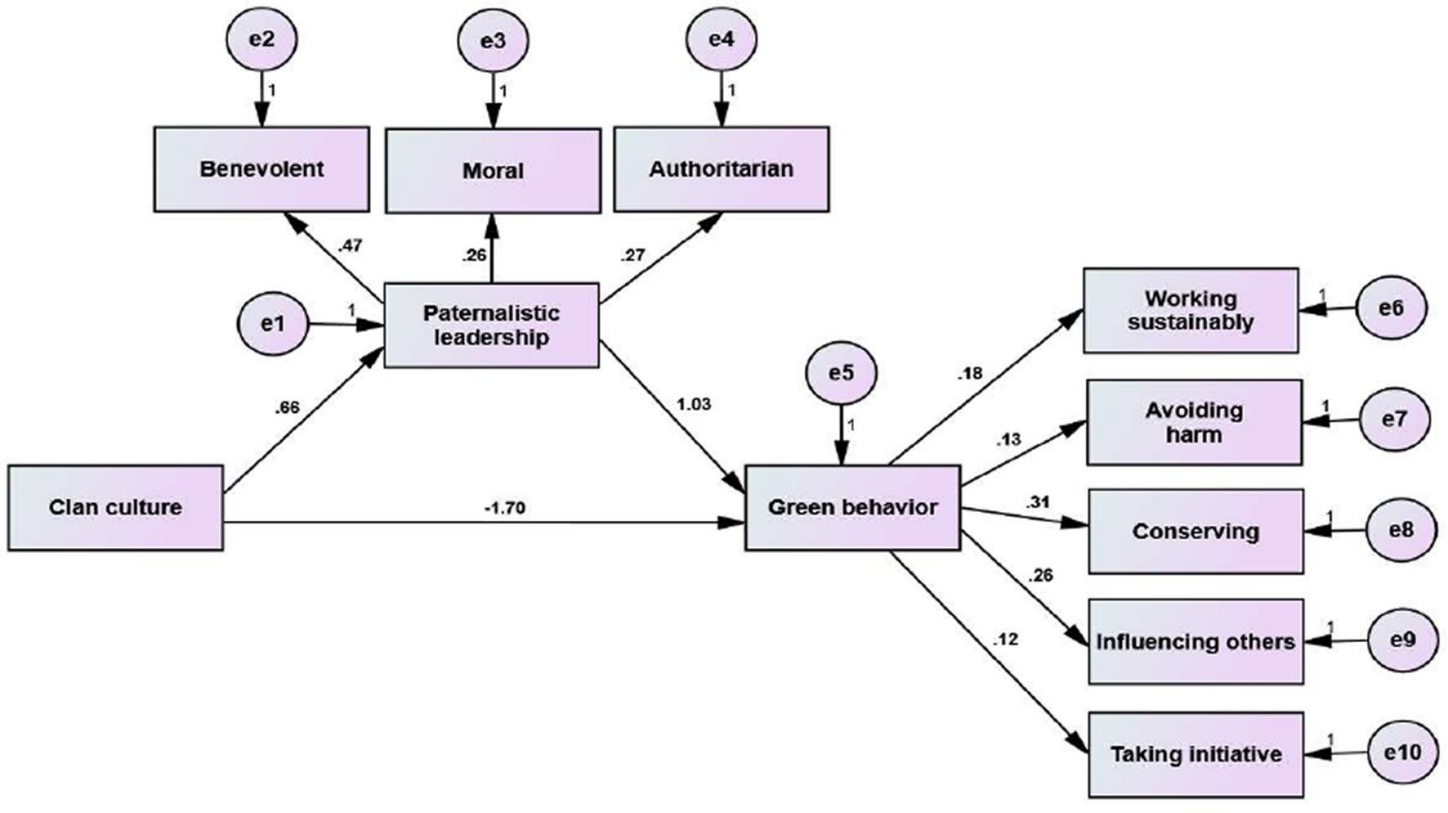
The mediating effect of paternalistic leadership on the relationship between clan culture and green behaviors among nurses (*n* = 780)

Table 4 provides an analysis of the direct and indirect effects in the mediating model, while Fig. 2 highlights the mediating role of paternalistic leadership in the relationship between clan culture and green behaviors among nurses. The direct effects reveal that clan culture has a significant negative direct effect on green behaviors (β = -1.70, *p* = 0.001), indicating that clan culture, on its own, may inhibit environmentally sustainable behaviors. Nevertheless, clan culture positively influences paternalistic leadership (β = 0.66, *p* = 0.001), suggesting that supportive and cohesive cultural environments foster a paternalistic leadership style. Additionally, paternalistic leadership positively affects green behaviors (β = 1.03, *p* = 0.001), emphasizing its critical role in promoting sustainability-related actions among employees. The indirect effect demonstrates that paternalistic leadership partially mediates the relationship between clan culture and green behavior, with a significant positive impact (β = 0.68, *p* = 0.001), indicating that leadership plays a key role in translating clan culture into green behaviors.

**Table 3:** Analyzing the correlation coefficients among the study variables (*n* = 780)

Variables	Clan culture	Benevolent	Moral	Authoritarian	Total paternalistic leadership	Working sustainability	Avoiding harm	Conserving	Influencing others	Taking initiative	Total green behavior
Benevolent (r)	0.575**										
Moral (r)	− 0.345**	0.270**									
Authoritarian (r)	0.310**	− 0.166**	− 0.611**								
Total paternalistic leadership (r)	0.399**	0.710**	0.367**	0.313**							
Working sustainability (r)	− 0.129**	0.287**	0.684**	− 0.175**	0.528**						
Avoiding harm (r)	− 0.376**	− 0.152**	0.523**	− 0.140**	0.153**	0.207**					
Conserving (r)	− 0.411**	0.059	0.650**	− 0.303**	0.243**	0.785**	0.430**				
Influencing others (r)	− 0.163**	0.410**	0.650**	− 0.458**	0.342**	0.421**	0.207**	0.255**			
Taking initiative (r)	− 0.244**	− 0.038	0.323**	− 0.197**	0.036	0.235**	0.037	0.366**	0.368**		
Total green behavior (r)	− 0.387**	0.197**	0.840**	− 0.401**	0.386**	0.782**	0.537**	0.831**	0.697**	0.551**	
Nurses’ loneliness (r)	− 0.267**	− 0.382**	− 0.344**	0.121**	− 0.394**	− 0.142**	− 0.068	− 0.043	− 0.278**	− 0.070	− 0.185**

r = Pearson correlation. *p- value is significant ˂ 0.05 level (2-tailed). **p- value is significant ˂ 0.01 level (2-tailed)

**Fig. 3:**
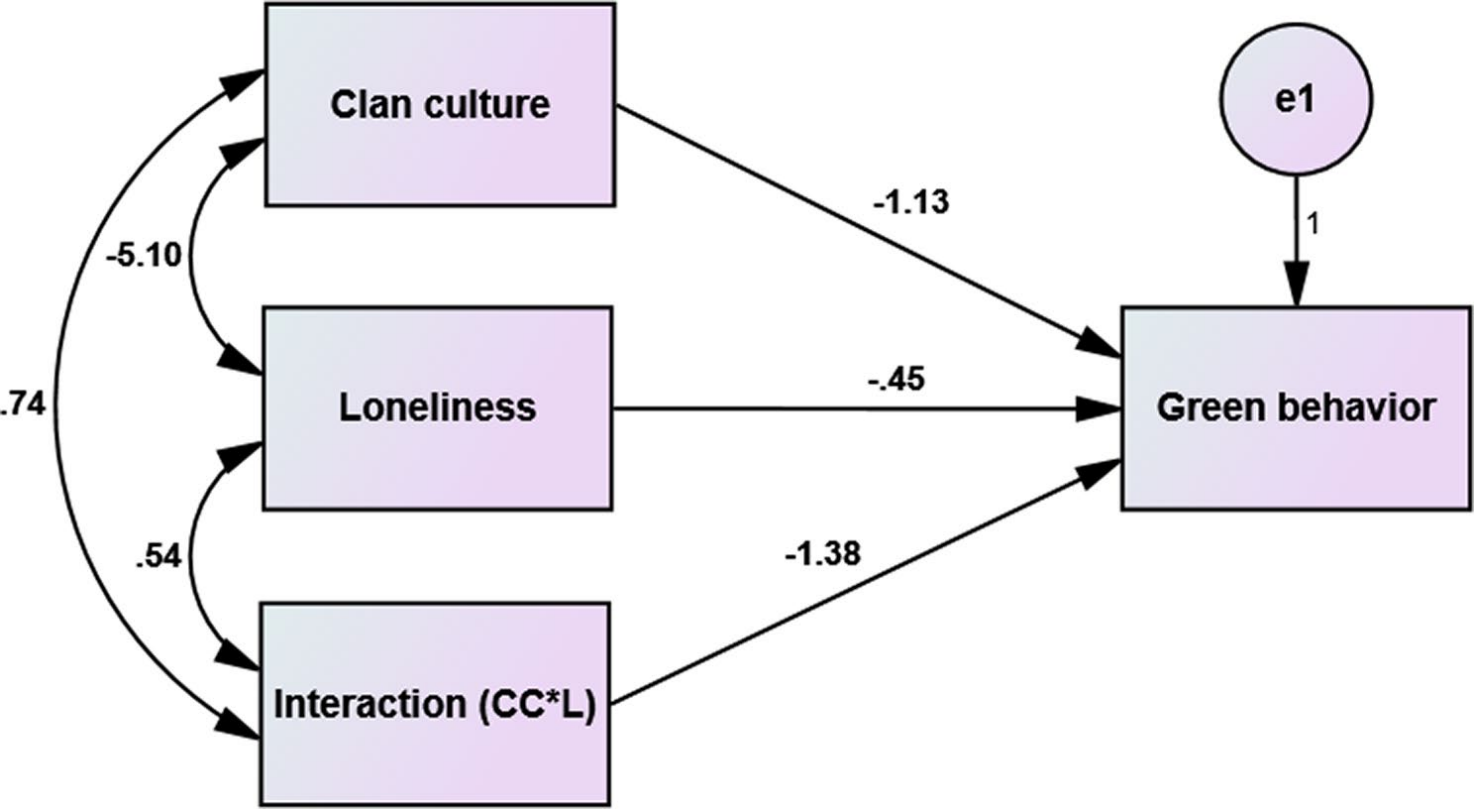
The moderating effect of loneliness on the relationship between clan culture and green behaviors among nurses (*n* = 780). CC = Clan culture. L = loneliness. Interaction referred to the moderator

Table 4; Fig. 3 present the moderating role of loneliness in the relationship between clan culture and green behavior. Loneliness negatively and significantly impacts green behavior (β = − 0.446, *p* = 0.002), signifying that individuals experiencing loneliness are less likely to engage in sustainable practices. The moderating effect of loneliness on the relationship between clan culture and green behavior is significant (β = -1.38, *p* = 0.007). This interaction suggests that the negative impact of clan culture on green behavior becomes more pronounced for individuals who experience higher levels of loneliness. Essentially, loneliness exacerbates the tendency for stronger clan cultures to discourage green behaviors, possibly because the individual’s sense of isolation within a group-driven culture makes it harder to engage with environmentally sustainable initiatives.

**Table 4:** Tests of direct and indirect effects of different study variables (*n* = 780)

Effects	β	BC 95% CI Lower/Upper	*P*
**Direct effects in the mediating model:**			
Clan culture → green behavior	− 1.70	− 1.800/- 1.603	0.001**
Clan culture → paternalistic leadership	0.66	0.564/0.756	0.001**
Paternalistic leadership → green behavior	1.03	0.956/1.097	0.001**
**The indirect effect in the mediating model**:			
Clan culture → green behavior through paternalistic leadership	0.68	0.587/0.780	0.001**
**The moderating effect of loneliness**:			
Interaction (clan culture * loneliness)	− 1.38	− 1.83/- 0.93	0.007**

*BC = Biased-corrected percentile method. CI = Confidence interval*. *p- value is significant ˂ 0.05 level. **p- value is significant ˂ 0.01 level

## Discussion

This study explored the interplay between clan culture, paternalistic leadership, and workplace loneliness in influencing nurses’ green behavior. The findings provide valuable insights into the mechanisms that promote environmentally sustainable practices in healthcare settings.

### Clan culture and green behaviors among nurses

The findings revealed that clan culture has a strong and significant negative direct effect on green behaviors among nurses. This result can be explained by several factors. First, clan cultures emphasize tradition and established practices, which can lead to resistance to change and limit the adoption of new sustainability practices. Second, the prioritization of patient care and team cohesion may overshadow environmental concerns, making green behaviors less important. Third, groupthink and social norms within a close-knit culture can discourage individuals from adopting eco-friendly practices if they are not widely accepted by the group. Lastly, the lack of external motivation, such as formal policies or rewards for sustainability, may result in less attention to green behaviors if they are not aligned with the team’s intrinsic values.

This finding is consistent with Kiptulon et al. [28], who examined the impact of organizational culture in healthcare services revealed that clan and hierarchy cultures notably reduced work-related stress and burnout, resulting in greater job satisfaction and work engagement. However, the study did not specifically examine green behavior, leaving its effect on environmental initiatives open to interpretation. Conversely, Atalla et al. [29], who examined the link between pro-social leader behaviors and nurses’ sustainability consciousness, revealed that organizational culture serves as a mediator and the culture that supports pro-social behaviors was linked to increased sustainability consciousness among nurses, indicating that specific cultural elements can encourage green behaviors.

### Clan culture and paternalistic leadership among nurses

The current study found that clan culture has a strong positive direct effect on paternalistic leadership. This outcome may be attributed to the alignment between clan culture and paternalistic leadership, as both emphasize strong leader-follower relationships built on trust and guidance. Clan culture promotes employee loyalty and commitment, making employees more receptive to a leader’s decisions. The acceptance of hierarchy in clan cultures reduces resistance to authority, allowing paternalistic leadership to be seen as legitimate rather than controlling. Additionally, the emphasis on shared values and ethical behavior supports moral leadership, while the culture’s focus on employee development ensures that mentorship and guidance are well received, enhancing overall well-being and growth. This outcome is in the same line with research by **Petek and Yeşiltaş** [5] which discovered that paternalistic leadership positively correlated with clan culture. Similarly, **Lee and Ding** [30] found that benevolent leadership aligns more effectively within clan cultures. On the other hand, **Jackson** [31] indicated that paternalistic leadership may be seen as overly authoritative or outdated in modern organizational environments, particularly in those that prioritize egalitarianism and employee autonomy. This perception can result in lower employee satisfaction and engagement, contrasting with the positive outcomes seen in traditional clan cultures. These studies emphasize that although clan cultures can facilitate paternalistic leadership, its effectiveness depends on cultural dynamics and shifting organizational values.

### Paternalistic leadership and green behaviors among nurses

The current study findings exposed that paternalistic leadership positively affects green behaviors. This outcome is likely due to several factors: Paternalistic leadership fosters trust and strong leader-follower relationships, encouraging nurses to align with sustainability goals. Leaders serve as ethical role models, inspiring green practices, while their support and mentorship boost employee engagement and satisfaction, motivating participation in environmental initiatives. This leadership style also promotes organizational loyalty, aligning nurses’ values with the organization’s green values. Lastly, paternalistic leadership reduces resistance to change by offering guidance and support, making the adoption of sustainability practices smoother and facilitating the integration of green behaviors into daily routines. Correspondingly, **Mi et al.** [11] revealed that both benevolent and authoritarian leadership directly promote various forms of employee green behavior, including task-related green behavior, eco-civic engagement, and eco-helping behaviors. Harmoniously, **Wang et al.** [32] concluded that benevolent leadership has a positive impact on employee green behavior, whereas authoritarian elements may lead to the opposite effect.

### Paternalistic leadership as a mediator

As regards the mediating role of paternalistic leadership; the study findings presented that paternalistic leadership partially mediates the relationship between clan culture and green behavior, with a significant positive impact. This finding highlights the importance of leadership in shaping sustainable practices within healthcare organizations. While clan culture provides a supportive environment, it is paternalistic leadership that can provide the direction and accountability needed to translate that support into actual green behaviors. Leaders who combine care and authority can effectively guide nurses to adopt eco-friendly practices and ensure that sustainability becomes an integral part of nursing care. Congruently, Peng et al. [33] determined that paternalistic leadership can shape the work environment, influencing employees’ experiences and behaviors. This is in line with the assertions made by Avolio et al. [34] that leadership is essential in converting cultural values into practicable actions. The significance of paternalistic leadership is further shown by the favorable effect it has on green behaviors. By establishing a sense of accountability, offering resources, and setting an example of sustainable practices, leaders who take a paternalistic approach are likely to promote green behaviors. The benign side of paternalistic leadership is probably what motivates its beneficial effects on green behavior, but Cheng et al. [26] warns that its authoritarian elements may inhibit creativity and engagement. This finding is supported by a study carried out by Robertson and Barling [35] who emphasized the importance of leadership in promoting sustainability. However, this finding diverges from a research by Hartnell et al. [36] which suggested that clan culture can indirectly hinder broader strategic goals when relational priorities dominate. These results demonstrate that paternalistic leadership can bridge this gap and effectively mediate the relationship between clan culture and green behaviors.

### Workplace loneliness as a moderator

The current study findings indicated that workplace loneliness moderates the relationship between clan culture and green behaviors. This suggests that loneliness exacerbates the already negative influence of clan culture on green behavior. In a culture that prioritizes relational harmony, individuals experiencing loneliness may feel alienated or disconnected, reducing their likelihood of engaging with group-driven initiatives like sustainability efforts. This result is consistent with the results of a study carried out by Hickethier et al. [37], who found that loneliness can diminish the impact of supportive organizational culture on employee performance and engagement, including green behavior. In the same way, Hartnell et al. [36] contended that the focus placed on social ties in clan culture may unintentionally alienate those who feel left out, increasing their disengagement. Clan culture’s relational emphasis may backfire in certain situations, escalating feelings of loneliness and decreasing general involvement in corporate objectives, such as green behavior.

The idea that clan culture naturally hinders eco-friendly conduct is contested by Rupp et al. [38], who stress that even relationally driven cultures can encourage green behaviors when sustainability is ingrained as a core goal inside the business culture. These results imply that whether or not sustainability is expressly valued in the culture may have an impact on the observed inverse link between clan culture and green behavior. Additionally, Laschinger et al. [39] highlighted the role of supportive leadership in mitigating loneliness and promoting engagement. This suggests that leadership styles, such as paternalistic leadership, may help counteract the adverse effects of loneliness within clan cultures, aligning relational priorities with sustainability goals. These studies demonstrated the complexity of the relationship between clan culture, workplace loneliness, and green behavior, highlighting that while a supportive culture is essential, workplace loneliness can hinder the effectiveness of cultural initiatives.

## Conclusion

This study underscores the importance of leadership in translating organizational culture into sustainable practices. While clan culture, on its own, may deprioritize green behaviors, paternalistic leadership serves as a critical mediator, enabling organizations to align relational harmony with environmental sustainability. By fostering supportive leadership and integrating sustainability into cultural values, organizations can address both relational and ecological goals.

### Limitations, future directions, and recommendations

The study relies on self-reported questionnaires, which may introduce response bias due to social desirability. To mitigate this, participants were assured of anonymity and confidentiality, and validated measurement scales with established reliability were used. Future research could enhance data accuracy by incorporating multi-source data collection methods, such as supervisor or peer evaluations, behavioral observations, and implicit measures.

Additionally, future studies should explore the specific impact of paternalistic leadership dimensions and assess the long-term sustainability of green behaviors fostered through leadership interventions. Furthermore, this study does not examine how different dimensions of loneliness (e.g., emotional vs. social) uniquely influence pro-environmental behavior. Future research should investigate these distinctions and explore tailored interventions to address loneliness and promote environmental behaviors across diverse organizational cultures.

By implementing the following recommendations, healthcare organizations can create a supportive environment, encourage leadership development, enhance nurses’ engagement in green behaviors, and reduce loneliness. (1) Training healthcare leaders to adopt a paternalistic leadership style can significantly impact the promotion of green behavior. (2) To maximize the benefits of paternalistic leadership, healthcare organizations should integrate green behavior policies with their cultural values, ensuring that the leadership is both supportive and aligned with sustainable practices. (3) Building a supportive work environment that promotes teamwork, communication, and a sense of belonging can help mitigate resistance to green behavior. (4) Healthcare organizations should prioritize addressing workplace loneliness through team-building activities, peer support systems, and promoting open communication. (5) Ensuring that all employees feel connected to their team and the organization’s mission can enhance the likelihood that they will engage in green behaviors. (6) Nurse leaders should be trained to recognize signs of workplace loneliness and take steps to create a supportive work environment.

### Implications for nursing management and leadership

To address these findings, organizations should prioritize enhancing clan culture while avoiding its potential drawbacks, promote benevolent leadership while reducing authoritarian tendencies, and implement targeted strategies to improve green behavior and mitigate workplace loneliness. Interventions could include: (1) Enhancing Clan Culture: Managers should promote a collaborative, family-like work environment, integrate sustainability into organizational values, and recognize green initiatives to encourage pro-environmental behavior. (2) Utilizing Paternalistic Leadership: Nurse leaders should lead by example, provide guidance and mentorship, and maintain fair and ethical leadership to strengthen nurses’ commitment to sustainability. (3) Reducing Workplace Loneliness: Implementing peer support programs, open communication, and well-being initiatives can enhance social connections and engagement in green behavior. (4) Implementing Policies and Training: Organizations should offer green training programs, establish sustainability committees, and integrate eco-friendly practices into performance evaluations to ensure long-term commitment. By adopting these strategies, nursing management can create a more sustainable, supportive, and environmentally responsible healthcare workforce.

## Data Availability

The data that support the findings of this study are available from the corresponding author upon reasonable request.
